# Predictors of the clinical severity of T1DM presentation at diagnosis in children and adolescents with type 1 diabetes mellitus (T1DM)

**DOI:** 10.1007/s42000-023-00518-7

**Published:** 2023-12-27

**Authors:** Kyriaki Karavanaki, Anastasia Korona, Spyridon Karanasios, Lydia Kossiva

**Affiliations:** Diabetic Clinic, 2nd Department of Pediatrics, ‘P. & A. Kyriakou’ Children’s Hospital, National and Kapodistrian University of Athens, Thivon & Levadeias Str, 115 27, Athens, Greece

**Keywords:** Childhood diabetes, Ketoacidosis, Autoimmunity

## Abstract

**Purpose:**

We aimed to assess factors associated with the presence and severity of ketoacidosis (DKA) at pediatric type 1 diabetes (T1DM) diagnosis, in relation to pancreatic, associated and familial autoimmunity.

**Methods:**

Antibodies against pancreatic beta-cells, organ specific autoantibodies (thyroid, celiac, and parietal) and family history of autoimmunity were retrospectively evaluated in 116 T1DM patients aged 11.9 ± 4.6 (mean ± SD) years, with disease duration 7.62 ± 3.67 years (mean ± SD).

**Results:**

Most patients (67.2%) presented with DKA at diagnosis. Younger children (< 2 years) had tenfold risk of DKA, compared to older children (12.1–15 years) (OR = 10.8, 95% CI: 1.0–116.9, *P* = 0.05). Fasting c-peptide levels were lower in the DKA group (OR = 0.26, 95% CI = 0.07–0.89, *P* = 0.033). The number of anti-pancreatic antibodies at disease onset did not show any significant correlations with the presence (*p* = 0.889) or severity of DKA (*p* = 0.863). All patients with multiple autoimmunity (> 2 autoimmune diseases plus T1DM) presented with DKA. Familial autoimmunity acted protectively against DKA manifestation (OR = 0.40, 95% CI = 0.16–1.0, *P* = 0.051).

**Conclusions:**

Among newly diagnosed T1DM patients, 67.2% presented with DKA. Younger age, lower c-peptide and the presence of associated autoimmunity were predictive factors of the presence and severity of DKA at diagnosis. High degree of suspicion, due to family history, may prevent DKA development and severity.

## Introduction

Approximately 65** × **10^**3**^children/year are diagnosed with T1DM and they most frequently present with diabetic ketoacidosis (DKA) as the initial manifestation [1]. DKA is reported to be the leading cause of death in children with T1DM and is accompanied by considerably lower c-peptide levels, reflecting lower residual beta cell function [2, 3]. In the long term, DKA is associated with poor diabetes control and possible neurological and cognitive deficits associated with persistent hypo- or hyperglycemia, as well as elevated health system costs due to prolonged hospitalization [4–7]. It is important to identify the factors predisposing to higher occurrence of DKA at T1DM diagnosis, and especially those that can be prevented. Most studies agree that factors associated with increased risk of DKA development at T1DM diagnosis include younger age, delayed diabetes diagnosis or treatment, lower socioeconomic status, lack of health insurance, lower body mass index, and preceding infection [8, 9]. On the other hand, family history of diabetes among first degree relatives, higher parental education, and higher incidence of T1DM in the country of residence are factors that seem to have a protective role, contributing to a timely diagnosis [8]. Regarding pancreatic autoimmunity status at disease onset and its correlation to severity of initial T1DM presentation, studies show controversial results. Pancreatic antibodies, mainly those against glutamic acid decarboxylase (GADA), tyrosine phosphatase-like insulinoma antigen 2 (IA2A), and islet cell antibodies (ICA), act as markers of beta-cell autoimmune destruction and their positivity level or concomitant presence of more than one antipancreatic antibodies are hypothesized to be related to a higher rate of DKA at diagnosis [9–11]. Other investigators, however, do not confirm such an association [12–15]. Similarly, the concomitant presence of associated autoimmunity in T1DM patients has not been clearly correlated to the severity of initial diabetes presentation. Parkkola et al. showed that children with an established autoimmune disease at T1DM diagnosis had a milder metabolic decompensation [16]. On the contrary, other studies have shown that celiac disease or hypothyroidism diagnosis before T1DM onset are associated with a more severe clinical manifestation and DKA frequency [17, 18]. Family history of diabetes seems to protect from severe initial manifestation of T1DM due to earlier recognition of symptoms. Studies evaluating the effect of additional autoimmune diseases other than diabetes, not only within the nuclear family but within the extended family as well, are scarce [16, 19–21]. To date, it has been shown that among the factors related to DKA identified at T1DM diagnosis, the most frequently reported include age of onset, followed by socioeconomic status and family history of T1DM; however, other precipitating factors may still be unrecognised. Thus far, limited data are available describing the effect of associated and familial autoimmunity on the severity of T1DM during initial presentation. The aim of the present study was to investigate how DKA presence and severity at T1DM diagnosis were related to the presence or absence of pancreatic, associated/familial autoimmunity **and to evaluate the aggravating role of younger age in T1DM first clinical manifestation severity, as previously reported.**

## Material and methods

### Study subjects

The study population consisted of 121 children, adolescents, and young adults (61 males) with T1DM treated at the Diabetic Clinic of the Second Department of Pediatrics of Children’s Hospital ‘P. & A. Kyriakou’ Athens, Greece, from January 2002 to December 2016. The age of the study participants ranged from 1 to 20 years, age at T1DM onset was 1–15 years, and disease duration ranged from 2 months to14.5 years. T1DM diagnosis was based on I.S.P.A.D. criteria (random plasma glucose ≥ 200 mg/dl and symptoms of diabetes, or fasting plasma glucose ≥ 126 mg/dl, or 2-h post-load glucose ≥ 200 mg/dl during an oral glucose tolerance test or HbA1c > 6.5%) [22]. Medical records were reviewed and patients with incomplete records were excluded from the study. The study was approved by the Ethics Committee of the ‘P&A Kyriakou’ Children’s hospital and was also in accordance with the Declaration of Helsinki (No: 293/12.01.2015), while written informed consent was obtained from the parents.

### Study design

This was a retrospective population study. Age at T1DM diagnosis and presence of pancreatic, associated, and familial autoimmunity were considered as risk factors, while severity of initial T1DM clinical presentation (DKA, DKA severity, and coma) was considered as the outcome. The patient’s age at T1DM diagnosis, fasting c-peptide levels, the presence and titer of GADA, and IA2A as indices of pancreatic autoimmunity as well as the presence of familial and associated autoimmunity were recorded and their correlations were analyzed. In our study population, only the above two pancreatic autoantibodies were initially estimated at T1DM diagnosis and, when absent, we proceeded with the estimation of ICA, endogenous insulin autoantibodies (IAA), and those against ZnT8, as suggested by Kawasaki et al. [23].

#### Severity of initial clinical presentation and laboratory findings at diagnosis

Evaluation of initial T1DM presentation was determined by the presence of DKA and its severity as well as the presence of coma. According to the I.S.P.A.D. guidelines, DKA was defined as pH < 7.30 or/and HCO3 < 15 mmol/lt. Depending on the severity of acidosis, DKA was defined as mild (pH < 7.3 or HCO3 < 15 mmol/lt), moderate (pH < 7.2 or HCO3 < 10 mmol/lt), or severe (pH < 7.1 or HCO3 < 5 mmol/lt) [24]. Coma was defined as Glasgow Coma Scale (GCS) < 8.

##### Associated autoimmunity

Associated autoimmunity was defined as the presence of one or more autoimmune diseases apart from T1DM in the same patient. Double autoimmunity was defined as the coexistence of T1DM with one autoimmune disease, while the presence of more than one autoimmune disease apart from T1DM was defined as multiple autoimmunity. The autoimmune diseases that were included in the patients’ questionnaire were the following: autoimmune thyroiditis, Graves’ disease, celiac disease, autoimmune gastritis, autoimmune hepatitis, Addison’s disease, multiple sclerosis, vitiligo, psoriasis, alopecia areata, chronic mucocutaneous candidiasis, juvenile idiopathic arthritis, sarcoidosis, and systemic lupus erythematosus. The above diseases and their combinations with T1DM are included in the autoimmune polyendocrine syndrome (APS) type I and ΙΙΙ [25]. The presence of antithyroid antibodies with normal TSH and fT4 values was characterized as thyroid autoimmunity, whereas the presence of antithyroid antibodies with abnormal TSH value and normal or abnormal fT4 values, or abnormal thyroid ultrasound scan findings was characterized as autoimmune thyroiditis. Respectively, the presence of anti-parietal cell antibodies (APCA) was defined as gastric autoimmunity, while the presence of relative clinical symptoms in addition to APCA positivity was classified as autoimmune gastritis. Finally, there is a distinct risk of diagnosed celiac disease and the presence of anti-tissue transglutaminase antibodies (anti-tTG) or/and antiendomysial antibodies (anti-EMA).

#### Familial autoimmunity

Familial autoimmunity was defined as the presence of at least one autoimmune disease, either within the nuclear family (first degree relatives, i.e., parents, siblings) or among the extended family (second- and third-degree relatives).

### Data collection

All data were acquired from the archives of the Diabetic Clinic of the Second Department of Pediatrics, University of Athens, ‘P.&A. Kyriakou’ Children’s Hospital, Athens, Greece. The documented parameters were as follows: (a) demographic data (gender, age at T1DM diagnosis, and current age), (b) disease duration, (c) weight, height, BMI, and their percentiles at first visit after diagnosis, (d) DKA at T1DM diagnosis and DKA severity, (e) fasting c-peptide levels and antipancreatic antibodies GADA and IA2A at diagnosis, (f) mean glycosylated hemoglobin A1c (HbA1c) during the last year of follow-up, (g) associated autoimmune diseases as well as the time of their diagnosis, clinical or subclinical course, and need for medical treatment, (h) autoimmune diseases among nuclear and extended family members, as reported by parents, and (i) presence of organ-specific autoantibodies, and specifically antibodies against 1. specific thyroid gland proteins (thyroglobulin anti-TG and thyroid peroxidase anti-TPO), 2. antibodies against intestinal mucosa (anti-EMA and anti-tTG), and 3. antibodies against gastric mucosa (APCA) at T1DM diagnosis and during follow-up.

Normal fasting c-peptide levels were considered as 1.77–4.68 ng/ml. Evaluation of antipancreatic antibodies GADA and ΙΑ2A was done using a radioimmunoassay (RIA) method, with an upper normal limit of 0.9 U/ml and 0.75 U/ml, respectively. Antithyroid antibodies were measured using an immunoluminometric assay (ILMA). They were considered positive when > 100 IU/ml for antiTG, and > 16 IU/ml for antiTPO. Subclinical thyroiditis was diagnosed with TSH levels 5–7 μIU/ml and at least one positive antithyroid antibody on two consecutive measurements or/and consistent ultrasound findings. Clinical autoimmune thyroiditis diagnosis was additionally based on TSH values greater than 7 μIU/ml, low FT4 levels or/and goiter. Antibodies against celiac disease (anti-tTG-IgA and anti-EMA-IgA) were measured using ELISA. Anti-tTG-IgA upper normal levels were considered as 20 units, while 20–30 units were considered as weakly positive. Moreover, total IgA levels were measured in all children. In cases of IgA deficiency, the diagnosis of celiac disease was suspected when high levels of IgG anti-tTG or anti-EMA antibodies were detected. When high levels (≥ 60 units) of anti-tTG-IgA or/and anti-EMA-IgA were detected in two consecutive measurements, together with consistent clinical symptoms, small intense biopsy was performed. Celiac diagnosis was based on biopsy typical findings. APCA were measured using indirect immunofluorescent assay (IFA), and a titer higher than 1:40 was considered positive. Endoscopy was performed when APCA were higher than 1:160, together with increased gastrin levels and/or clinical symptoms of gastritis, or in the case of suspicion of H. pylori infection. Regarding HbA1c, mean value during the past 12 months of follow-up was calculated and normal values were considered to be between 4.6 and 6.2%. HbA1c was measured in capillary blood with a DCA 2000 + analyzer (Bayer Corporation).

### Statistical analysis

Continuous variables are presented as mean value ± SD if normally distributed and as median value (interquartile range, IQR) if not normally distributed. Normal distribution was assessed using the Kolmogorov Smirnov test. The chi-aquare test and Fisher’s exact test were used for categorical variables. In cases of borderline statistically significant results, further control was carried out with univariate regression analysis. For the comparison between quantitative and qualitative variables with two categories, Student’s t-test was used for variables with normal distribution and the non-parametric Mann Whitney U test for variables not following a normal distribution. The Kruskal–Wallis test was used to compare quantitative and qualitative variables in more than two groups. To test linear correlation, Spearman’s rho test was applied. Finally, to assess the overall effect of the factors under examination related to the probability of DKA, univariate logistic regression analysis was initially carried out, followed by multifactorial logistic regression analysis in order to evaluate the adjusted odds ratio. Analysis was also performed by age group, categorized in two models, as follows: (i) a) ≤ 5 years old, b) 5.1–10 years old, and c) > 10 years old, and (ii) a) ≤ 2 years old, b) 2.1–12 years old, and c) > 12 years old. This categorization was based on literature reports, as many studies have shown that children aged 2—5 years old show experience of a more severe clinical course at T1DM presentation [8, 21, 26–28]. Gender, c-peptide levels, and associated autoimmunity were regarded as potential confounders and adjusted by multivariate modeling. The level of significance was set at *P* = 0.05.

## Results

### Demographic and laboratory characteristics

Of the 121 children included in the study, 61 were male **(50.4%).** Mean age ± SD was 11.99 ± 4.63 years (range: 2.0–20.0), age at T1DM diagnosis was 7.62 ± 3.67 years (range: 1.0–15.0), and median disease duration was 3.5 years (range: 2 months to 14.5 years). GADA antibodies titer ranged from 0 to 2888 U/ml and IA2A titer ranged from 0.1 to 3960 U/ml. Associated autoimmunity was present in **25.6%** of the patients, while familial autoimmunity within the nuclear and/or the extended family was present in 62.8% of our patients. Mean ± SD HbA1c during the last year of follow-up was 7.66 ± 1.16%.

At T1DM diagnosis, mean pH ± SD was 7.21 ± 0.15 (range: 6.83–7.46) and median HCO_3_ was 9 mmol/lt (range: 0.90–29.0). Seventy-eight patients (78/121, **64.5%**) presented with DKA. Of those, **15.7%** (19/121) had mild DKA, **22.3%** (27/121) had moderate, and **26.5%** (32/121) had severe DKA. Thus, more than half of our patients (**48.8%**, 59/121) presented with moderate or severe DKA at T1DM diagnosis. Median C-peptide at T1DM diagnosis was 0.59 ng/ml (IQR: 0.45–0.86).

### Factors related to the presence and severity of DKA at T1DM diagnosis

#### DKA and age at T1DM diagnosis

**Fig. 1:**
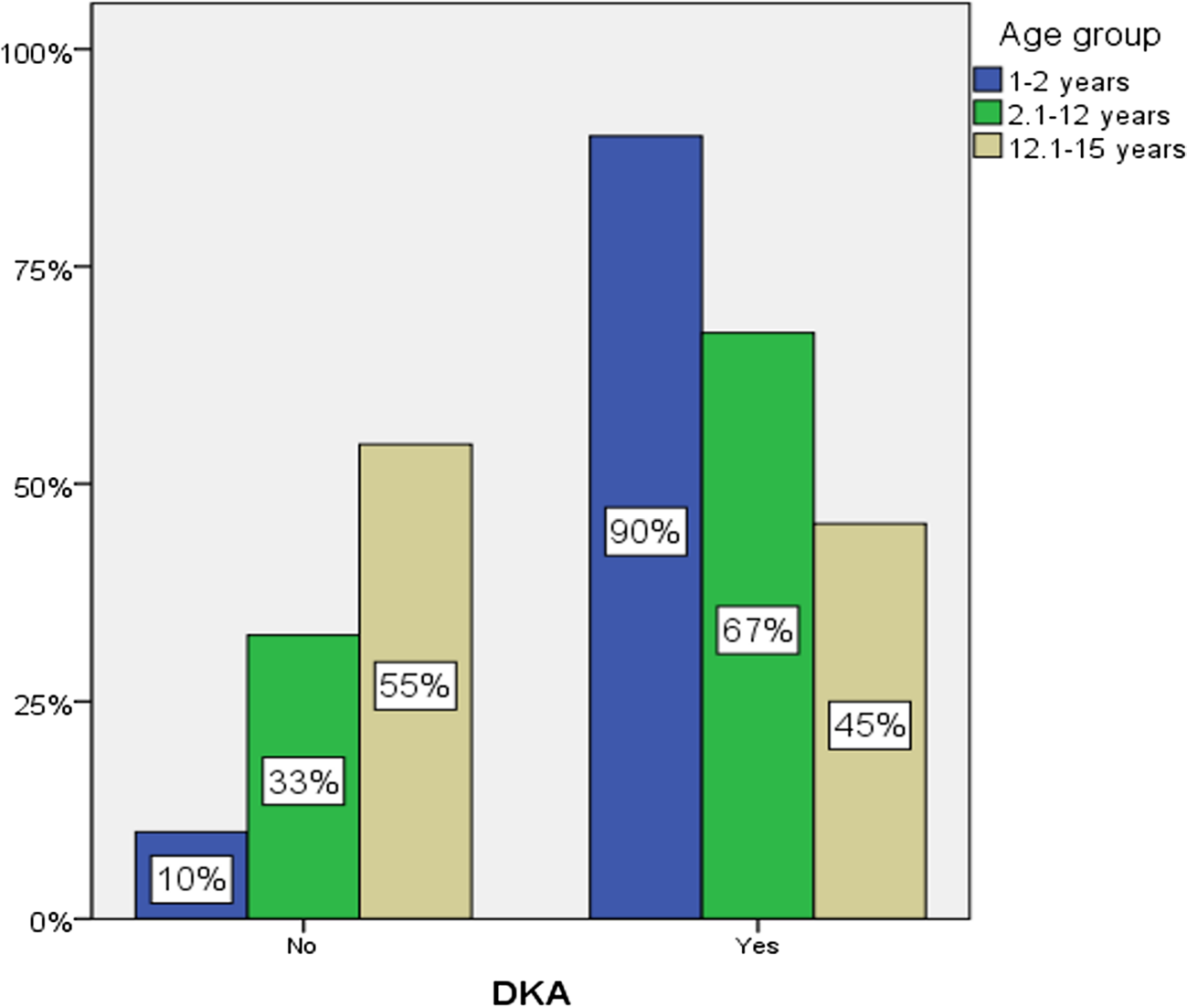
DKA frequency by age group

**Fig. 2:**
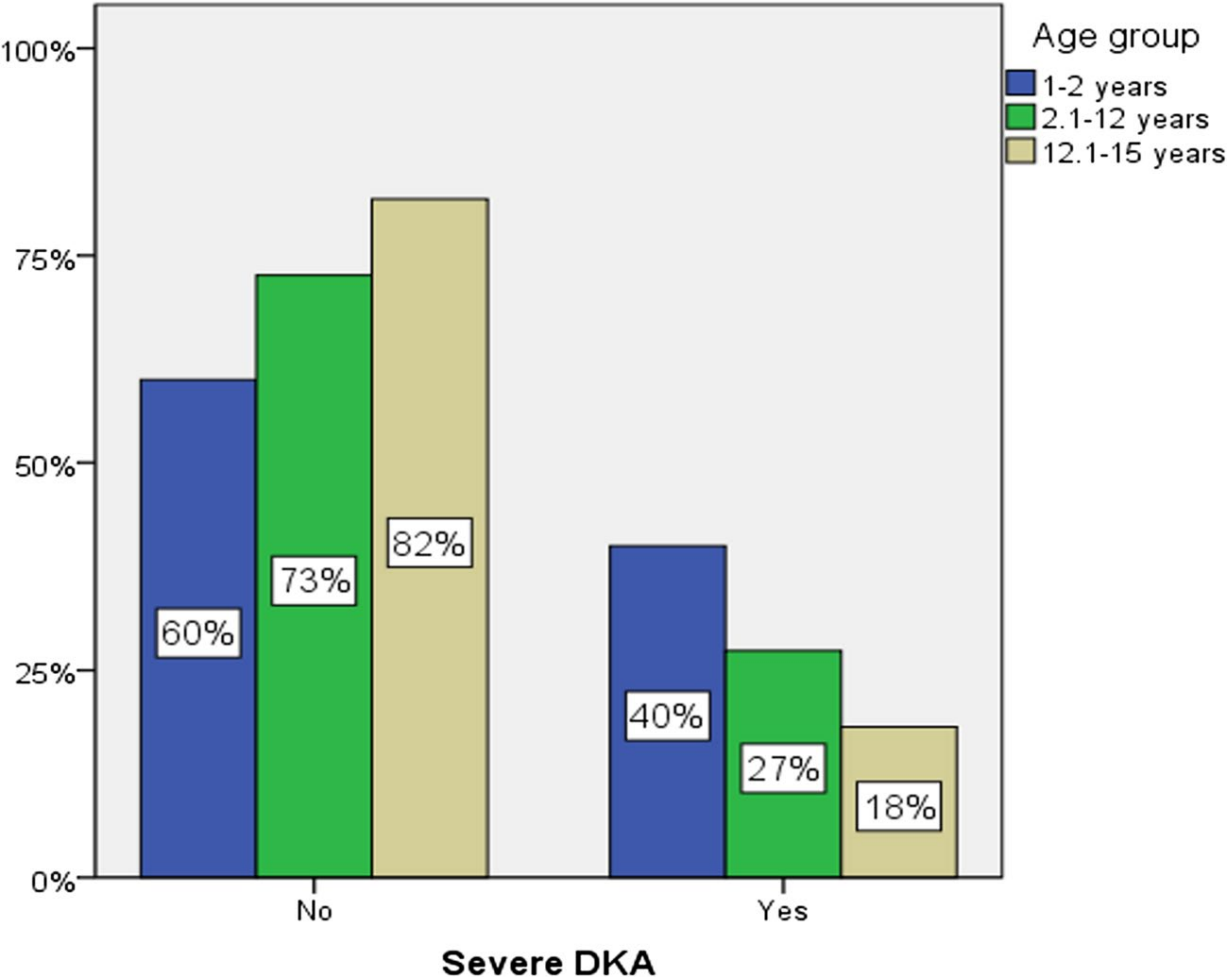
Severe DKA frequency by age group

DKA frequency was highest (90%) in the youngest age group (1–2 years old) and lowest in the 12.1–15 years old group (45%) (Fig. 1). Almost half of the patients in each age group had severe DKA, with the highest prevalence (40%) in the youngest age group and the lowest (18%) in the oldest age group (Fig. 2). Statistical analysis of DKA frequency by age group did not yield statistically significant results.

#### Comparison of patient groups presenting with or without DKA at T1DM diagnosis

C-peptide levels of patients with DKA at T1DM diagnosis were marginally non-significantly lower than those without (0.56 vs 0.70 ng/ml, *p* = 0.055, Table 1). The presence of DKA at diabetes diagnosis was significantly lower in patients with a family history of autoimmunity in the extended family than those without (35.9 vs 53.5%, *p* = 0.024). There were no other significant differences among examined variables between the two groups with or without DKA at diagnosis.

**Table 1:** Association of clinical and laboratory factors with the presence or absence of DKA at T1DM diagnosis in children and adolescents

	Presence of DKA	Absence of DKA	*P*
Age at diagnosis (years)	7.3 ± 3.6	8.42 ± 3.61	0.143
Gender (male/female)	38/40 (48.7/51.3)	23/20 (53.5/46.5)	0.508
c-peptide (ng/ml)	0.56 (0.43–0.80)	0.70 (0.51–1.00)	0.055
GADA or IA2 presence (yes/no)	56/6 (90.3/9.3)	30/4 (88.2/11.8)	0.739
GADA titer (U/ml)	4.05 (0,9–27)	7.18 (0.20–20.79)	0.560
IA2 titer (U/ml)	4.83 (0,7–12,4)	4.00 (0.70–18.40)	0.665
Associated autoimmunity at diagnosis (yes/no)	9/69 (11.5/88.5)	8/35 (18.6/81.4)	0.562
Associated autoimmunity during follow-up (yes/no)	21/57 (26.9/73.0)	12/31 (27.9/72.0)	0.945
Familial autoimmunity – extended family (yes/no)	28/50 (35.9/64.1)	23/20 (53.5/46.5)	0.024

Quantative variables are referred to as mean value ± SD or as median value (IQR), and qualitative as Ν (%). DKA: diabetic ketoacidosis, GADA: glutamic acid decarboxylase antibodies. IA2A: protein tyrosine phosphatase-like protein IA2 antibodies

#### Comparison of DKA severity at T1DM diagnosis with different parameters

Table 2 shows the comparison of clinical and laboratory study parameters between patients with severe DKA and those with the following: (a) mild/moderate DKA or (b) without DKA. Patients with severe DKA at diabetes diagnosis were significantly younger than those without DKA (6.69 vs 8.42 years, *p* = 0.048, Student’s t-test). Fasting c-peptide levels at diabetes diagnosis were not significantly different between groups with different degrees of DKA severity or absent DKA (Table 2). C-peptide levels were significantly lower in younger children with DKA than older ones (0.52 vs 0.75 ng/ml, *P* = 0.040), and especially in those with moderate or severe DKA (0.38 vs 0.65 ng/ml, *P* = 0.034).

**Table 2:** Association of DKA severity at T1DM diagnosis with clinical and laboratory parameters

	Severe DKA	Mild/Moderate DKA	P	Severe DKA	Absence of DKA	P
Age at T1DM diagnosis (yrs)	6.69 ± 3.55	7.83 ± 3.69	0.177^a^	6.69 ± 3.55	8.42 ± 3.61	0.048^a^
Gender (male/female)	17/15 (53.1/46.9)	21/25 (45.7/54.3)	0.516^b^	17/15 (53.1/46.9)	23/20 (53.5/46.5)	0.858^b^
c-peptide (ng/ml)	0.57 (0.41–0.79)	0.54 (0.43–0.84)	0.867^c^	0.57 (0.41–0.79)	0.70 (0.51–1.00)	0.103^c^
GADA/IA2 positivity (yes/no)	22/1 (95.7/4.3)	34/5 (87.2/12.8)	0.398^d^	22/1 (95.7/4.3)	30/4 (88.2/11.8)	0638^d^
GADA titer (U/ml)	4.90 (1.07–42.0)	3.98 (0.80–10.3)	0.337^c^	4.9 (1.0–42.0)	7.18 (0.20–20.79)	0.247^c^
IA2 titer (U/ml)	6.07 (0.9–13.87)	4.60 (0.14–12.46)	0.526^c^	6.07 (0.9–13.9)	4.00 (0.70–18.40)	0.643^c^
Associated autoimmunity at diagnosis (yes/no)	3/29 (9.4/90.6)	6/40 (13.0/87.0)	0.730^d^	3/29 (9.4/90.6)	8/35 (18.6/81.4)	0.494^d^
Associated autoimmunity at follow-up (yes/no)	7/25 (21.9/78.1)	14/32 (30.4/69.6)	0.402^b^	7/25 (21.9/78.1)	12/31 (27.9/72.0)	0.666^b^
Familial autoimmunity (yes/no)	21/11 (65.6/34.4)	25/21 (54.3/45.7)	0.319^b^	21/11 (65.6/34.4)	27/16 (62.8/37.2)	0.988^b^
Familial autoimmunity in extended family (yes/no)	10/22 (31.3/68.8)	18/28 (39.1/60.9)	0.475^b^	10/22 (31.3/68.8)	23/20 (53.5/46.5)	0.044^b^

Quantitative variables are expressed as mean ± SD or median (IQR) and qualitative ones as Ν (%). *DKA* Ddiabetic ketoacidosis, *GADA* Gglutamic acid decarboxylase autoantibodies, *IA2* protein tyrosine phosphatase-like protein IA2

^a^Student’s t-test

^b^Pearson’s chi-Square

^c^Mann-Whitney Ttest

^d^Fisher’s exact test

The presence of severe DKA was significantly less frequent in patients with familial autoimmunity in the extended family (31.3 vs 53.5%, *P* = 0.044) (Table 2). No significant difference in the presence/absence of associated autoimmunity at T1DM diagnosis or during follow-up according to the presence or severity of DKA at diagnosis was observed (Table 1, 2); however, all six patients with multiple autoimmunity (T1DM and > / 2 additional autoimmune diseases presented with DKA at diagnosis.

#### Familial autoimmunity

Most T1DM patients in our study (62.8%) had familial autoimmunity. Specifically, 24.8% of our patients had one relative with autoimmune disease in their nuclear and/or extended family, 23.1% had two relatives, 11.6% had three, 1.7% had four, and 1.7% had five relatives. In total, the most frequent familial autoimmune disease was Hashimoto’s thyroiditis in almost half of the patients’ relatives (49.5%), while the second most frequent autoimmune disease in the family was T1DM, with 22.3% of our patients having at least one relative with T1DM. The prevalence of other autoimmune diseases in the patients’ families was much lower, as follows: juvenile rheumatoid arthritis (4.1%), vitiligo (4.1%), idiopathic inflammatory bowel disease (3.3%), psoriasis (3.3%), celiac disease (0.8%), and Graves’ disease (0.8%).

#### Univariate and multivariate logistic regression analysis

Univariate analysis showed that the likelihood of the development of DKA at T1DM diagnosis was ten times higher in children \ < 2 years than in the older age groups (OR = 10.80, *p* = 0.050) (Table 3). Moreover, the likelihood of severe DKA was 20 times greater in the youngest age group (children \ < 2 years) (OR: 21.42, 95% CI: 1.55–295.63, *P* = 0.022) and five times greater in children \ < 5 years old compared to those aged 10.1–15 years (OR: 4.69, 95% CI: 1.36–16.13, *P* = 0.014). Fasting c-peptide levels were significantly lower among children with DKA at diagnosis; in particular, for every reduction of c-peptide levels by one unit the likelihood of DKA increased by 74% (OR: 0.26, 95% CI: 0.08–0.90, *P* = 0.033), while adjustment for age did not alter the association. Finally, the presence of a family history of T1DM prevented the development of DKA (OR:0.25, CI: 0.09–0.64, *P* = 0.004) and there was a significant association with the number of relatives with T1DM (OR = 0.27, *P* = 0.002) (Table 3). Specifically, patients with a family history of T1DM in the extended family had 75% less likelihood of presenting with DKA at diabetes diagnosis in comparison with those without a family history of T1DM, while for every additional relative with T1DM the risk for DKA at diabetes diagnosis decreased by 73%. The rest of the factors in Table 3 were not associated with DKA at T1DM presentation.

**Table 3:** Univariate logistic regression analysis of the factors that are associated with the presence/identification of DKA at T1DM diagnosis

	OR	95% CI	*P*
Sex (male/female)	1.30	0.59–2.83	0.509
Age of T1D diagnosis (yrs)	0.92	0.82–1.02	0.143
Age category of T1D diagnosis (yrs)			
12.1 – 15			
1–2	10.80	1.00–116.99	0.050
2,1–12	2.47	0.70–8.75	0.159
c-peptide	0.26	0.07–0.89	0.033
GADA	1.00	0.99–1.00	0.829
IA2	0.99	0.99–1.00	0.386
Associated autoimmunity at diagnosis (yes/no)	0.69	0.22–2.12	0.523
Associated autoimmunity (yes/no)	1.03	0.42–2.48	0.945
Number of associated autoimmune diseases	1.33	0.66–2.67	0.421
Familial autoimmunity (extended family) (yes/no)	0.45	0.20–0.99	0.049
Number of relatives in the extended family	0.61	0.39–0.96	0.035
Family history of T1D (nuclear and extended) (yes/no)	0.25	0.09–0.64	0.004
Number of relatives with T1D (nuclear and extended)	0.27	0.12–0.63	0.002
Family history of T1D (nuclear family) (yes/no)	0.26	0.06–1.17	0.080

*GADA* Gglutamic acid decarboxylase autoantibodies, *IA2* protein tyrosine phosphatase-like protein IA2

*OR: Oodds ratio

**CI:95% confidence interval

Following adjustment for gender and associated autoimmunity, multivariate logistic regression analysis (Table 4) revealed two factors independently related to the presence of severe DKA at T1DM diagnosis, as follows: (i) age at disease onset and (ii) the number of relatives with autoimmune disease in the extended family. Children with severe DKA were significantly younger at diagnosis compared to children without DKA (6.69 ± 3.55 vs 8.42 ± 3.61 years, *P* = 0.048). A lower risk of severe DKA was observed among patients with autoimmunity in the extended family (OR = 0.40, *P* = 0.050). The association was stronger with an increasing number of relatives with an autoimmune disease (OR = 0.52, *p* = 0.010) (Table 4).

**Table 4:** Multivariate logistic regression analysis of the factors related to DKA at T1DM diagnosis

	Adjusted OR*	95% CI**	*P*
Age at T1D diagnosis (yrs)			
1–2	21.42	1.55–295.63	0.022
2.1–12	2.65	0.70–9.97	0.149
Gender	1.39	0.59–3.24	0.442
Associated autoimmunity (yes/no)	1.20	0.45–3.16	0.713
Number of relatives with autoimmunity (extended family)	0.52	0.32–0.85	0.010
Familial autoimmunity (extended family) (yes/no)	0.40	0.16–1.00	0.051

*CI* confidence interval

*OR:adjusted odds ratio

**95%

## Discussion

In the present study, we investigated the factors associated with DKA presence and severity at T1DM diagnosis in children and adolescents, along with the possible role of pancreatic, associated, and familial autoimmunity. **Studies assessing the effect of pancreatic and associated autoimmunity on T1DM presentation and severity have shown inconsistent results, while, to the best of our knowledge, only one previous study has examined the effect of familial autoimmunity on clinical severity at diabetes diagnosis** [16].

In our study, **64.5%** of children presented with DKA at T1DM diagnosis and **48.8%** with moderate or severe DKA. According to a systematic review including data from 31 countries, DKA frequency at T1DM diagnosis worldwide ranges from 12.8 to 80%. The highest rates are reported in the United Emirates (80%) and the lowest in Sweden (12.8%) [1]. This may be due to timelier diagnosis in countries with a high prevalence of T1DM.

Children presenting with DKA in the present study were youngand those with severe DKA even younger. Specifically, the likelihood of severe DKA was five times greater in children < 5 years old compared to those aged 10.1–15 years (*P* = 0.014), and 20 times greater in children < 2 years (*P* = 0.022). Similarly, previous studies have highlighted young age (< 5 or < 2 years) as the most important risk factor for DKA, particularly severe DKA [8, 21, 26–29]. However, another study by Hekkala et al. reported that older children also had a higher frequency of DKA and severe DKA at T1DM diagnosis (10–14.99 years) [27]. High frequency of DKA in young children may be attributed to a lower index of suspicion, atypical clinical presentations, and underdeveloped compensatory biological mechanisms, leading to rapid metabolic derangement, especially on account of a concurrent infection [8, 21]. An additional factor could be faster and more aggressive destruction of pancreatic beta-cells due to genetic or environmental factors during the first years of life. As for adolescents older than 12 years, puberty seems to be an aggravating factor for DKA, since pubertal hormones lead to increased insulin resistance and inadequate parental supervision may lead to underassessment of symptoms [30]. **Another factor associated with DKA presence and severity is delayed T1DM diagnosis **[28]**. This was confirmed during the recent COVID-19 pandemic during which time parental hesitance to visit hospitals due to fear of contracting SARS-CoV2 limited parental understanding of disease severity and obstacles to access healthcare services contributed to increased frequency and severity of T1DM presentation **[31].

In patients with T1DM, different endotypes have recently been described based on demographic (age at diagnosis and race/ethnicity), genetic, immunological, histopathological, metabolic, and clinical characteristics [32, 33]. Therefore, and as supported by our findings, young age at T1D diagnosis may represent a specific endotype, associated with a smaller number of islets, low c-peptide levels at diagnosis and duration of remission and higher frequency of DKA at diagnosis, and a greater number of antipancreatic and coexistent autoantibodies. This endotype has also been strongly associated with the presence of HLA DR3/DR4 and excess mortality [33, 34].

In agreement with previous reports [12, 35, 36], fasting c-peptide levels were significantly lower in younger children with DKA (*P* = 0.040), and especially those with moderate or severe DKA (*P* = 0.034). DKA at T1DM diagnosis is associated with lower endogenous insulin secretion, expressed by lower c-peptide levels, and is more frequent among young children and those with delayed diagnosis [37]. Furthermore, according to the TEDDY (The Environmental Determinants of Diabetes in the Young) study, prompt T1DM diagnosis is associated with higher fasting c-peptide levels and lower DKA frequency, regardless of age. Therefore, DKA manifestation is affected by time to diagnosis and age at diagnosis [38, 39].

In our study, the presence and severity of DKA at T1DM diagnosis was not associated with the number and titers of the pancreatic autoantibodies GADA and IA2A. Pancreatic autoantibodies constitute an index of autoimmune process resulting in the destruction of pancreatic beta-cells [10]. The role of pancreatic autoimmunity in relation to T1DM clinical presentation and DKA is controversial. According to some reports, the presence of GADA at diabetes onset is associated with more severe and faster destruction of pancreatic beta-cells, while its persistence in serum for a long time possibly plays a protective role, as it may indicate the presence of residual beta-cells stimulating their production [9–11, 40]. As in our study, others did not confirm a strong link between DKA and pancreatic autoimmunity [12, 41]. Fajardo et al. reported that adults with positive autoantibodies, especially GADA, had DKA at T1DM diagnosis more frequently, whereas in children, pancreatic autoimmunity did not appear to correlate with diabetes clinical presentation and DKA [15].

Thus, other factors beyond pancreatic autoimmunity, such as T1DM endotypes, might affect the severity of clinical presentation of T1DM and life-long glycemic metabolic control. A specific endotype among patients with T1DM has been described based on HLA alleles and the first detected autoantibody. Thus, GADA autoantibodies emerge as a sole marker of autoimmunity for children above 6 years of age and adult patients with LADA [33] and are strongly associated with the HLA-DR3 haplotype, slower disease progression, and specific immunological characteristics [42]. In contrast, in T1DM children, another endotype has been described based on the presence of insulin autoantibodies (IAA). These autoantibodies are the sole marker of pancreatic autoimmunity in patients below 2 years of age, are strongly linked to the HLA DR4 haplotype, and are associated with faster disease progression, hyperimmune CD20 insulitis, and higher frequency of DKA at diabetes diagnosis [33]. **Since IAA autoantibodies were not measured in the present study, we may have missed IAA positivity, particularly among young patients with severe clinical presentation. Inversely, by measuring GADA and IA2, we identified older patients with slower disease progression **[33]**, which might explain the absence of association between pancreatic autoimmunity and the severity at T1DM presentation in this study.**

In this study, no significant difference in the severity of clinical presentation of diabetes was observed among the groups of patients according to the presence and the number of associated autoimmune diseases. However, all six patients with multiple autoimmunity (i.e., T1DM and > /2 additional autoimmune diseases) presented with DKA at diagnosis. It is possible that a high level of autoimmunity reserve, probably indicating genetic susceptibility, leads to a more aggressive initial clinical presentation of diabetes. In contrast, Parkkola et al. found that children with a pre-existing autoimmune disease at the time of T1DM diagnosis had lower glucose levels and milder acidosis than children without associated autoimmunity, regardless of age. This finding was attributed by the authors to a slower evolution of the disease in those children rather than earlier diagnosis [16].

In this study, we found that a history of autoimmunity (and especially T1DM) in the extended family had a protective effect on the risk of DKA at T1DM diagnosis, and this effect increased with the increasing number of relatives with autoimmune diseases. / The latter suggests that autoimmunity in the extended family results in earlier recognition of diabetes symptoms, this hypothesis being partially in agreement with previous studies [16, 21]. One could argue that family history of T1DM, suggestive of a genetic predisposition to diabetes, might also affect the age and severity of clinical presentation of the disease. However, our findings suggest increased awareness of the family and of the primary care clinician regarding early diabetes symptoms lead to earlier diagnosis and prevention of DKA development.

The limitations of our study include its retrospective nature and the limited number of participants. Nevertheless, the study group was representative of the Greek population with childhood diabetes as it consisted of both children admitted to our hospital with new onset T1DM symptoms with or without DKA, and children referred from regional hospitals for evaluation. In addition, we measured only two antipancreatic antibodies (GADA and IA2A), which had been reported as the best predictors of future development of T1DM [43]. As previously suggested, patients with newly diagnosed diabetes should be initially tested for the presence of GADA and IA2A and, if negative, further testing for ICA, IAA, and ZnT8 autoantibodies should be pursued [23]. Finally, familial autoimmunity was based on reports without antibody measurements.

The advantages of the present study include a thorough investigation of various factors associated with the development and severity of DKA at diabetes diagnosis, including pancreatic, associated, and familial autoimmunity, the latter being a novelty of the study. Our findings could help increase clinicians’ awareness of the value of early T1DM diagnosis in the prevention of DKA.

In conclusion, DKA was frequent among newly diagnosed children with T1DM (64.5%), with half of them (48.8%) having moderate or severe DKA. In our study, younger age at diagnosis, lower c-peptide levels, and the presence of associated autoimmunity were predictive factors of the presence and severity of DKA at T1DM presentation. On the other hand, familial autoimmunity was a protective factor of DKA. However, the possible impact of associated and pancreatic autoimmunity on the pathogenesis of DKA at T1DM first presentation remains unclear. Clarification and evaluation of the exact role of the aforementioned factors, especially for the definition of specific T1DM endotypes, may lead personalized patient management and the application of specific immune therapies for the prevention of islet cell destruction and disease progression. Additionally, public campaigns to raise awareness of T1DM symptoms among parents, teachers, and healthcare professionals may lead to earlier diagnosis and a subsequent decrease in DKA frequency and severity.
